# Synchronous Malignant Otitis Externa and Squamous Cell Carcinoma of the External Auditory Canal

**DOI:** 10.1155/2013/837169

**Published:** 2013-09-30

**Authors:** R. Y. Chin, T. B. V. Nguyen

**Affiliations:** Department of Otolaryngology Head and Neck Surgery, Nepean Hospital, The University of Sydney, Sydney, NSW 2747, Australia

## Abstract

*Objectives*. To discuss the management of a squamous cell carcinoma in the presence of malignant otitis externa. *Study Design*. We present only the third reported case in the literature of a synchronous tumour with malignant otitis externa in the literature. *Methods*. A case report and review of malignant otitis externa and squamous cell carcinomas of the external auditory canal are discussed. *Results*. A 66-year-old female is presented here with a 2-month history of a painful, discharging left ear refractory to standard antibiotic therapy. Computerised tomography, magnetic resonance imaging, technetium 99 m, and gallium citrate Ga67 scans were consistent with malignant otitis externa. Biopsy in the operating theatre revealed a synchronous squamous cell carcinoma of the external auditory canal. Primary resection of the tumour and surrounding tissues was performed with concomitant treatment with intravenous antibiotics. *Conclusions*. This is only the third case to be reported in the literature and highlights several important diagnostic and management issues of these two rare conditions. Both conditions may present in a similar manner on clinical assessment and radiological investigations. Aggressive management with surgical resection and treatment with appropriate intravenous antibiotics is necessary to give the best chance for cure.

## 1. Introduction

Malignant otitis externa (MOE) is a rare and potentially fatal invasive infection of the skull base. It can arise as a result of an infection of the external auditory canal “malignant otitis externa,” the middle ear, and sinusitis and as a complication of surgery of the skull base [1–3]. Although a variety of organisms can cause MOE, the predominant organism responsible for the infection is *Pseudomonas aeruginosa*, although fungal species such as *Aspergillus* may also be involved [3]. Squamous cell carcinomas (SCC) arise from the skin of the external auditory canal and are relatively rare entities. They are most common in individuals in their 6th decade of life and may be related to chronic infection, radiation exposure, and sun or cold exposure. Both squamous cell carcinomas of the external auditory canal and malignant otitis externa can present with a painful, discharging ear with granulation tissue involving the external canal refractory to initial antimicrobial therapy [4]. This case highlights the importance of a multidisciplinary team approach to both the diagnosis and treatment of these rare pathologies and reinforces the importance of biopsy in the diagnosis of two diseases, which are virtually indistinguishable on clinical, laboratory, or radiological grounds.

## 2. Materials and Methods

### 2.1. Case Report

**Figure 1:**
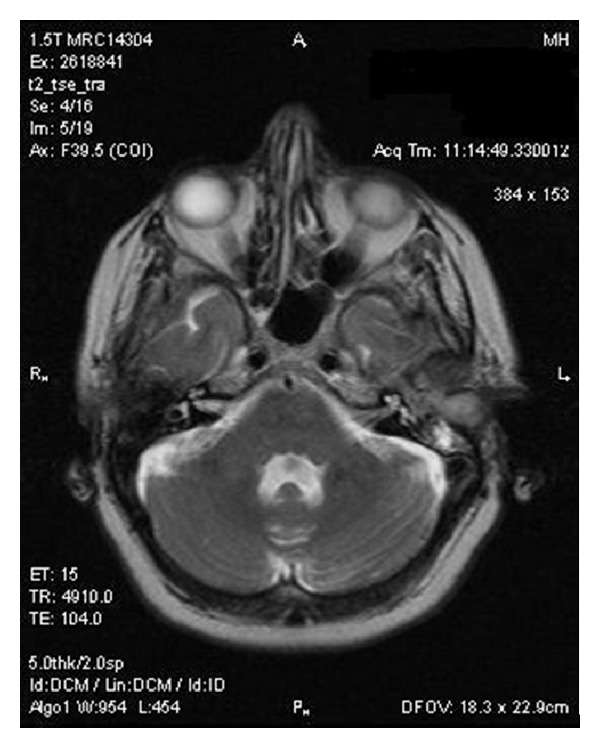
Axial T2 weighted MRI image demonstrating the left sided tumour mass.

A 66-year-old deaf lady presented with a 2-month history of left sided otalgia and discharge. This ear pathology was refractory to standard treatment of otitis externa, which included ear toilet with dry suction, pain control, and topical antibiotics. The initial trauma appeared to have been caused by cleaning the external canal with a cotton wool bud. Physical examination revealed an inflamed left external auditory canal with polypoid tissue obstructing much of the canal at the approximate level of the bony cartilaginous junction. Examination of her cranial nerves was unremarkable. Ear aspirates revealed a heavy growth of *Pseudomonas aeruginosa* and a moderate growth of *Staphylococcus aureus*. Her white cell count (WCC) and C-reactive protein were elevated at 11.3 × 10^3^/*μ*L, and 44.6 mg/L respectively. She was not diabetic or immunosuppressed. Computerised tomography (CT) scan showed soft tissue density involving the external auditory canal with bone erosion of the tegmen consistent with inflammatory destruction (Figure 1). There was also increased attenuation of the left side mastoid antrum and air cells consistent with mastoiditis. Given these initial findings, a provisional diagnosis was made of malignant otitis externa arising from an infection of the external auditory canal. Theatre time was allocated for her to have the external canal biopsied and toileted. Infectious disease and radiology were notified and she was commenced on intravenous synthetic penicillin (ticarcillin clavulanate potassium) and a quinolone (Ciprofloxacin) via a peripherally inserted intravenous catheter (PICC) line. An MRI scan and a technetium 99 m (Tc99) and Gallium citrate (Ga67) were organised. Both the Ga67 and Tc99 were consistent with malignant otitis externa involving the petrous temporal bone and mastoid process. 

Examination in theatre revealed granulation tissue and polyps along the entire length of the external auditory canal and predominant growth of *P. aeruginosa*. Histopathology demonstrated an inflamed stratified squamous epithelium with dysplasia and cellular atypia. Within this, there were embedded irregular nests of atypical squamous cells infiltrating the underlying stroma, and a diagnosis of moderately differentiated SCC was made.

**Figure 2:**
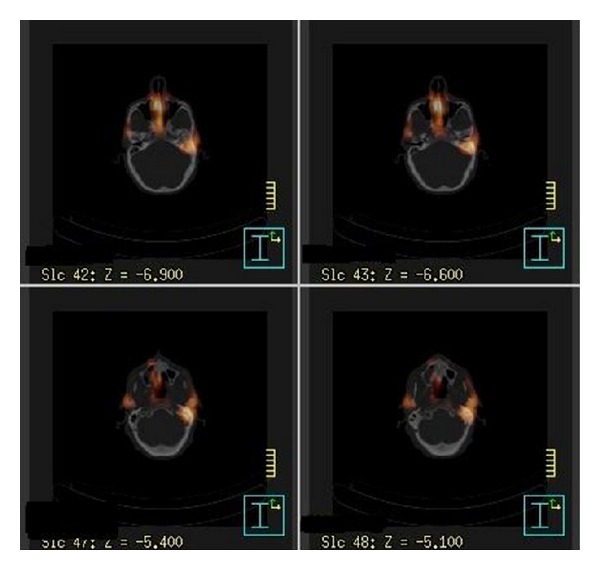
SPECT scan demonstrating increased areas of uptake in the region of the left petrous temporal bone and external auditory canal.

The patient was notified of the two separate diagnoses and a plan was made to treat both. The malignant otitis externa was to be treated in the standard manner with long-term intravenous and oral antibiotics. A positron emission tomography scan (PET) scan showed no evidence of metastatic disease and was also correlated with CT and MRI findings (Figure 2). The tumour was treated with a lateral temporal bone resection, parotidectomy, selective neck dissection, and reconstruction with a rectus abdominis muscle free flap and split skin graft. Intraoperatively, the tumour extended from the external canal to infiltrate the entire middle ear cavity with extension down into the eustachian tube, hypotympanum, tympanic facial nerve, and the oval window. The tumour was adherent to the middle cranial fossa dura. As the facial nerve was involved, it was segmentally resected from 2 mm distal to the geniculate ganglion to the stylomastoid foramen, with a sural nerve interposition graft repair. Involved dura was resected with a dural patch and temporalis muscle flap repair. Frozen section margins were all clear of disease. Final tumour stage was T4 N0 M0 disease. Otolaryngology head and neck surgery, plastic surgery, and neurosurgery performed the resection and reconstruction. The patient underwent a course of radiotherapy to the affected areas. Total treatment was 60 GY. 

On subsequent followup there has been no recurrence of the disease, and approximately 3 months of intravenous antibiotic therapy along with the lateral temporal bone resection appear to have cured the malignant otitis externa.

## 3. Discussion

MOE is a rare, invasive infection which typically begins as a chronic infection of the external auditory canal (necrotising otitis externa), middle ear, and sinuses or may be as a result of skull base procedures [1–3]. It evolves from a superficial infection of the soft tissues to involve the deeper structures such as bone and cartilage and can be associated with cranial nerve impairment [3]. It is a disease best managed by a multidisciplinary team with primary involvement of otolaryngology head and neck surgery, infectious disease and radiology. 

It typically arises in patients who are immunosuppressed. Most commonly this immunosuppression takes the form of advanced age, diabetes mellitus, acquired immunodeficiency syndrome (AIDS), iatrogenic immunosuppression, or immunosuppression due to haematological abnormalities or malnourishment [3]. The latter group is more likely to affect children who have this disease [5]. 

The predominant organism in MOE is *P. aeruginosa*. The infection can be polymicrobial with other organisms such as *Aspergillus* spp (*fumigatus*), *S. aureus*, *Proteus mirabilis, Klebsiella oxytoca, Burkholderia cepacia, Candida* spp, *Scedosporium apiospermum*, *Pseudallescheria boydii,* and *Malassezia sympodialis*. The majority of fungal MOE occurs in immunosuppressed individuals with AIDS [3]. 

There has been a slow evolution of the treatment of MOE. Advances in antimicrobial therapy and the introduction of multidisciplinary care and long-term antibiotic therapy have seen this disease change into one which carried a high mortality of approximately 50% with frequent recurrence often requiring a major surgical intervention, to one with a relatively low mortality, and virtually no surgical intervention other than biopsy and debridement of necrotic tissue [3]. The introduction of parenteral semisynthetic penicillins and the fluoroquinolones is largely responsible for the decreased mortality of this disease [3].

Involvement of the ear and lateral skull base by squamous cell carcinoma is usually the result of a cutaneous neoplasm that originates from the skin of the pinna or the external auditory canal. Ultraviolet light exposure or thermal injury (cold) and radiation exposure and chronic infection are thought to predispose patients to this disease [4]. Rarely, squamous carcinomas can arise from the middle ear from metaplastic middle ear mucosa and are associated with chronic otitis media and human papilloma virus [6]. Treatment of squamous carcinomas of the external canal should be aggressive because of the high rate of recurrence. Treatment with en-block resection, selective neck dissection, and radiotherapy is recommended in cases like the one presented here, as recurrence rates and nodal metastasis are relatively high.

The authors present only the third case in the literature of a synchronous malignancy and malignant otitis externa [7, 8]. Both malignant otitis externa and squamous cell carcinomas of the external auditory canals are rare entities, and it is even rarer to have both occurring at the same time. Both pathologies present in a remarkably similar manner—clinically, radiologically, and on laboratory investigations [3, 4]. 

Clinically, both conditions often present with a painful, discharging ear refractory to standard treatment regimes of ear toilet and antibiotic therapy. Both conditions may be present with cranial nerve palsies, trismus, and lymphadenopathy. 

Radiologically, both conditions on CT and MRI may have abnormalities of the external auditory canal, soft tissue, and fluid within the middle ear and mastoid cavity, eustachian tube, and parapharyngeal space with or without concomitant bony destruction [6]. There are no studies in the literature which look specifically at Tc99 and Ga67 scanning in SCC of the external auditory canal, but it is conceivable that both these investigations would be positive in the presence of an extensive neoplastic process with bone erosion and chronic infection of the soft tissues without malignant otitis externa being present.

This case and the other cases reported in the literature [7, 8] highlight the importance of ear toilet and biopsy in the investigation and diagnosis of malignant otitis externa when the cause is a chronic infection of the external auditory canal (malignant or necrotizing otitis externa) refractory to standard treatment regimes. Successful diagnosis and management of both these pathologies involve a multidisciplinary team approach and a meticulous unification of a detailed clinical examination, laboratory investigations, appropriate radiology, and biopsy to create a complete clinical picture.

## References

[1] Slattery WH, Brackmann DE (1996). Skull base osteomyelitis: malignant external otitis. *Otolaryngologic Clinics of North America*.

[2] Grobman LR, Ganz W, Casiano R, Goldberg S (1989). Atypical osteomyelitis of the skull base. *Laryngoscope*.

[3] Grandis JR, Branstetter BF, Yu VL (2004). The changing face of malignant (necrotising) external otitis: clinical, radiological, and anatomic correlations. *Lancet Infectious Diseases*.

[4] Moody SA, Hirsch BE, Myers EN (2000). Squamous cell carcinoma of the external auditory canal: an evaluation of a staging system. *American Journal of Otology*.

[5] Rubin J, Yu VL (1988). Malignant external otitis: insight into pathogenesis, clinical manifestations, diagnosis, and therapy. *American Journal of Medicine*.

[6] Marioni G, Altavilla G, Busatto G, Blandamura S, De Filippis C, Staffieri A (2003). Detection of human papillomavirus in temporal bone inverted papilloma by polymerase chain reaction. *Acta Oto-Laryngologica*.

[7] Grandis JR, Hirsch BE, Yu VL (1993). Simultaneous presentation of malignant external otitis and temporal bone cancer. *Archives of Otolaryngology*.

[8] Mattucci KF, Setzen M, Galantich P (1986). Necrotizing otitis externa occurring concurrently with epidermoid carcinoma. *Laryngoscope*.

